# Effect of Overfeeding Shetland Pony Mares on Embryonic Glucose and Lipid Accumulation, and Expression of Imprinted Genes

**DOI:** 10.3390/ani11092504

**Published:** 2021-08-26

**Authors:** Nicky M. M. D’ Fonseca, Charlotte M. E. Gibson, David A. van Doorn, Ellen Roelfsema, Marta de Ruijter-Villani, Tom A. E. Stout

**Affiliations:** 1 Department of Clinical Sciences, Faculty of Veterinary Medicine, Utrecht University, 3584 CM Utrecht, The Netherlands; chrlt.gibson@gmail.com (C.M.E.G.); d.a.vandoorn1@uu.nl (D.A.v.D.); e.roelfsema@uu.nl (E.R.); M.Villani@uu.nl (M.d.R.-V.); t.a.e.stout@uu.nl (T.A.E.S.); 2 Department of Population Health Sciences, Faculty of Veterinary Medicine, Utrecht University, 3584 CL Utrecht, The Netherlands

**Keywords:** mare, epigenetic reprogramming, nutrient transporters, equine conceptus, maternal nutrition

## Abstract

**Simple Summary:**

In pregnant individuals, maternal overnutrition is associated with disturbances in the expression of specific genes and nutrient transporters in the early embryo, which can affect both fetal and placental development and have lasting effects on the health of resulting offspring. To examine how maternal overfeeding affects the equine embryo, Shetland pony mares were fed either a high-energy (HE: 200% of net energy requirements) or maintenance (control) diet. Mares from both groups were inseminated, and day-seven embryos were recovered and transferred to recipients from the same or the alternate group. The expression of several genes, nutrient transporters and DNA methyltransferases (DNMTs; play an important role in regulating gene expression) were determined in extra-embryonic membranes after recovery on day 28 of gestation. The expression of nutrient transporters was also assessed in endometrium recovered from recipient mares immediately after embryo removal. In addition, glucose uptake by day-28 extra-embryonic membranes, and lipid droplet accumulation in day-seven embryos were assessed. Maternal overfeeding resulted in elevated expression of several genes, DNMTs and nutrient transporters following embryo transfer from an HE to a control mare. The expression of two amino acid transporters was also elevated in the endometrium after embryo transfer from HE to control. Maternal overfeeding did not affect lipid droplet accumulation in day-seven embryos, or glucose uptake by membranes of day-28 embryos. It remains to be seen whether the alterations in gene expression are maintained throughout gestation and into postnatal life.

**Abstract:**

Maternal overfeeding is associated with disturbances in early embryonic epigenetic reprogramming, leading to altered expression of imprinted genes and nutrient transporters, which can affect both fetal and placental development and have lasting effects on the health of resulting offspring. To examine how maternal overfeeding affects the equine embryo, Shetland pony mares were fed either a high-energy (HE: 200% of net energy requirements) or maintenance (control) diet. Mares from both groups were inseminated, and day-seven embryos were recovered and transferred to recipients from the same or the alternate group. The expression of a panel of imprinted genes, glucose and amino acid transporters, and DNA methyltransferases (DNMTs) were determined in conceptus membranes after recovery on day 28 of gestation (late pre-implantation phase). The expression of nutrient transporters was also assessed in endometrium recovered from recipient mares immediately after conceptus removal. In addition, glucose uptake by day-28 extra-embryonic membranes, and lipid droplet accumulation in day-seven blastocysts were assessed. Maternal overfeeding resulted in elevated expression of imprinted genes (*IGF2, IGF2R, H19, GRB10, PEG10* and *SNRPN*), DNMTs (*DNMT1* and *DNMT3B*), glucose (*SLC2A1*), fructose (*SLC2A5*) and amino acid (*SLC7A2*) transporters following ET from an HE to a control mare. Expression of amino acid transporters (*SLC1A5* and *SLC7A1*) was also elevated in the endometrium after ET from HE to control. Maternal overfeeding did not affect lipid droplet accumulation in blastocysts, or glucose uptake by day-28 membranes. It remains to be seen whether the alterations in gene expression are maintained throughout gestation and into postnatal life.

## 1. Introduction

Research into the developmental origins of health and disease (DOHaD) has revealed the far-reaching effects that both maternal under- and overnutrition can have on the epigenome of the developing embryo and fetus, with the potential to influence susceptibility to lifestyle-related diseases (e.g., hypertension and type 2 diabetes) into adulthood [1]. Overfeeding is an increasingly common issue in horses and is currently a topic of interest primarily because of its association with an increased risk of metabolic syndrome [2] in the individual overfed animal. Equine metabolic syndrome is the collective term for a group of associated clinicopathological changes that includes obesity, insulin dysregulation (ID) and other related endocrine disturbances, and which together predispose to the development of endocrinopathic laminitis [2]. A less well-investigated area of study is the possibility for transgenerational effects and, in particular, whether maternal overfeeding or obesity in the periconception period affects the epigenome of the horse embryo and may predispose the offspring to the development of metabolic or orthopedic disease later in life [3].

Epigenetics refers to changes in gene expression that occur without any change in the DNA sequence itself, for example inhibition of gene transcription by methylation of the DNA or acetylation of the binding histones [4]. During gametogenesis and preimplantation development, epigenetic reprogramming must occur to ensure that the zygote/early embryo is capable of giving rise to all the cell types required to produce a viable embryo, fetus and neonate. During the initial period of reprogramming, nearly all of the epigenetic marks accumulated by the parental genomes are erased, e.g., by global DNA demethylation, with the exception of the parentally imprinted marks, such that the early blastomeres become totipotent [5]. The process of re-acquiring epigenetic marks, also known as DNA methylation reprogramming (DMR), then starts at or directly before blastocyst formation (day 5–7 after ovulation in the horse) [6], when cells begin to choose lineage fates, differentiate and lose potency [5,7]. The way in which embryonic demethylation and subsequent re-methylation occurs is influenced by the environment in which it occurs. In particular, in other species, maternal nutrition has been shown to influence epigenetic reprogramming, with the potential for lifelong effects on gene expression in the offspring, including increased susceptibility to complex, lifestyle related diseases in adulthood [8].

DMR also plays an important role in regulating the expression of imprinted genes [4]. Genomic imprinting is an epigenetic modification that involves inactivation of one of the two alleles of a gene in a parent-of-origin specific manner [9]. In general, imprinted genes are important for fetoplacental development and help regulate the nutrient supply to the fetus by influencing the size, architecture and nutrient transfer capacity of the placenta [10]. In this respect, it has been proposed that imprinted genes play a critical role in maternal-fetal resource allocation to balance maternal needs against fetal demands [9]. Based on this theory of a conflict for resources, maternally expressed genes, such as *IGF2R* and *GRB10* [11,12,13], are expected to limit conceptus growth, whereas paternally expressed genes, such as *PEG10* [14], should enhance fetoplacental growth [9]. *IGF2* and *IGF2R* have further been proposed to be key regulators of early placental formation, which in turn determines the capacity for fetal and placental growth during the remainder of gestation [13]. Murine studies have revealed profound consequences of maternal overnutrition and obesity on the expression of imprinted genes [15,16], suggesting, for example, that pre-conception exposure to a high-fat (HF) diet may influence re-programming of imprinted marks during early embryonic development. Besides the potential effects on embryonic epigenetic reprogramming, maternal diet clearly has the capacity to influence embryonic and fetal nutrient provision throughout gestation. In the horse, definitive placental formation, characterized by interdigitation of the chorioallantois with the endometrium, only starts at around day 40 of gestation, indicating that the capacity for uptake of nutrients during the first part of gestation is limited [17]. The equine conceptus is entirely dependent on histotrophic nutrition during the first 40 days of development [18]. Histotroph is synthesized by the endometrial glands and luminal epithelial cells and secreted into the uterine cavity; this “uterine milk” contains nutrients such as amino acids, carbohydrates, and lipids, together with growth factors, cytokines, and hormones [19]. The composition of the histotroph is determined by factors including the prevailing maternal endocrine milieu (e.g., circulating progesterone and estrogen concentrations) [20], endometrial nutrient transporters and, presumably, maternal circulating nutrient concentrations. The conceptus membranes also express specific transporters to facilitate the uptake of nutrients present in the histotroph into the embryo [18,21], and the expression of certain nutrient transporters may, in turn, be adjusted in response to the abundance of nutrients in the uterine cavity. For example, rodent and mice studies show increased expression of various amino acid and glucose transporters in the trophectoderm or placenta (respectively, day 18.5 and day 21) of mice fed an HF [22] and rats fed a HSHF [23] diet, when compared to control chow-fed mice or rats. Placental capacity to transfer glucose and amino acids to the fetus has also been shown to be enhanced in response to a maternal diet high in simple sugars and saturated fats [16].

The aim of this study was to determine the effect of maternal overfeeding on early epigenetic reprogramming and pathways for conceptus nutrient uptake. Mares were fed either a high-energy (HE) diet equating to 200% of net energy requirements, which resulted in gradual onset of obesity and ID [24], or a control maintenance diet. The effect of maternal overfeeding on pre-implantation conceptus development was investigated by transferring day-seven embryos (i.e., immediately after blastocyst formation) between HE and control mares and assessing the expression of genes coding for various imprinted genes and DNMTs, glucose and amino acid transporters in the conceptus membranes at day 28 of gestation (i.e., late in the pre-implantation period). To further assess the effects of maternal overfeeding on embryonic nutrient handling, the lipid droplet content was compared between day-seven embryos originating from control versus HE donor mares, and glucose uptake was examined in the extra-embryonic membranes of day-28 conceptuses, resulting from transfer of embryos between mares from the same or the alternate feeding group.

## 2. Materials and Methods

### 2.1. Horses and Husbandry

The study was performed between February 2014 and October 2016. Eighteen nulliparous Shetland pony mares (aged 2–9 years; body condition score (BCS) of 4–6/9 [25]; starting weight of 174 ± 19 kg) were monitored over periods ranging from 1–3 breeding seasons, based on their having and maintaining adequate fertility (Table 1). The breed was chosen because of its genetic predisposition to ID [26]. Mares were initially assigned randomly to either the control (*n* = 9) or an HE feeding (*n* = 9) group; HE mares participated for a maximum of two consecutive seasons due to the risk of developing laminitis after prolonged overfeeding. Groups were allowed access to a sand paddock every other day to enable social contact and limited exercise throughout the study. General health of all mares was assessed daily by monitoring heart rate, rectal temperature and gait. The ID status of the HE mares was examined via periodic oral glucose tolerance testing, which was performed 3 to 4 times per study year and reported in a previous study [24]. The experimental protocol was approved by the Committee on Animal Welfare of Utrecht University (DEC 2014.III.01.004).

### 2.2. Diet

**Figure 1:**
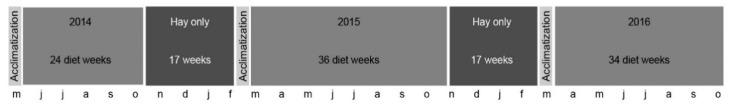
Study years 2014–2016 with the corresponding number of weeks that the diet was fed, and the corresponding calendar months (starting in May 2014) below the figure.

The composition of the diet has been described previously [24]. In brief, the diet of both the control and HE groups consisted of a concentrate feed (36% sugar + starch, 13% fat), grass hay (9% sugar, 2% fat), and a feed supplement to ensure adequate provision of minerals, trace elements, and vitamins (Pavo Vital Complete; Pavo, Boxmeer, The Netherlands). Control mares were fed 100% of their daily NE requirements set by the Centraal Veevoederbureau (4) (approximately 0.348 megajoule NE × BW^0.75^) to maintain a moderate body condition throughout the study. High-energy mares were fed 200% of their NE requirements to induce weight gain, as previously described by Carter et al. [27] with minor modifications. Energy intake was adapted to weight gain throughout the experiment to maintain a 200% NE intake. The experimental diet was fed during the periods of reproductive cyclicity and embryo collection, which accounted for 24 weeks in 2014, 36 weeks in 2015 and 34 weeks in 2016 (Figure 1). During the winter rest periods, to avoid exacerbating any incipient metabolic disbalance, all mares were fed a hay-only diet of at least 100% NE requirements. All mares had free access to water and a salt lick (KNZ^®^; Hengelo, The Netherlands). Hay and concentrate were fed in multiple meals per day at 08:00, 13:00 and 17:00 h.

### 2.3. Body Weight

All ponies were weighed weekly using a calibrated scale (Epelsa BCN100M: Grupo Epelsa, Madrid, Spain) to monitor weight gain.

### 2.4. Embryo Transfer, Conceptus and Endometrium Collection

During early estrus, mares were examined by ultrasonography every other day. Once a dominant follicle exceeded 30 mm in diameter, mares were examined daily until ovulation occurred. When a follicle reached ≥35 mm in diameter, donor mares were inseminated with fresh semen (minimum of 500 × 10^6^ sperm cells) from a single fertile stallion. Insemination was repeated every two days until ovulation occurred. To aid synchronization of estrus, mares were administered cloprostenol (Genestranvet^®^ 37.5 mg im) during diestrus and human Chorionic Gonadotrophin (Chorulon 1000 IE iv, Intervet Nederland B.V., Boxmeer, The Netherlands) when they were in estrus with a preovulatory follicle. Recipient mares followed the same procedure but were not inseminated. On day 7 after ovulation, embryos were collected by uterine lavage (3 L Lactated Ringer’s Solution, supplemented with 0.5% (*v*/*v*) heat-inactivated fetal calf serum), performed as described previously [28]. Embryos were then washed 5 times and transferred to holding medium (Syngro; Bioniche Animal Health, Pullman, WA, USA), their diameter was measured using a stereomicroscope (Olympus SZ-ST; Olympus, Tokyo, Japan) equipped with an eye-piece micrometer, and their quality was assessed on a scale of 1–4 [29]. Only good quality (grade 1–2) embryos were transferred to a recipient mare, or they were used for fluorescent staining.

At ET + 21 (day 28 of gestation), conceptuses were recovered by video-endoscopically guided collection after puncture of the membranes and aspiration of the yolk sac or allantoic fluid, as described previously [30]. After recovery, day 28 conceptuses were washed 3–4 times in large volumes of NaCl. The yolk sac (YS) and allantochorionic (AC) membranes were microscopically separated from each other and from the embryonic body using microsurgical scissors [30], and the different tissue samples were placed in holding medium at 37 °C (for 2-(N-(7-nitrobenz-2-oxa-1,3- diazol-4-yl)amino)-2-deoxyglucose [2-NBDG] live cell staining) or snap frozen in liquid nitrogen and stored at −80 °C (for PCR). After conceptus removal, endometrium samples were collected from the pregnant uterine horn of the recipient mare using an alligator forceps (Kruuse; Langeskov, Denmark).

To determine whether embryonic phenotype was affected by a change in maternal nutritional status embryos were transferred within and between groups as follows (Table 1):

- Control donor to control recipient mare (C-C);
- HE donor to HE recipient mare (HE-HE);
- Control donor to HE recipient mare (C-HE);
- HE donor to control recipient mare (HE-C).

### 2.5. Gene Expression

#### 2.5.1. RNA Extraction and cDNA Synthesis

To examine the effect of maternal diet on the expression of selected candidate genes in day-28 conceptus membranes, four day-28 conceptuses from each group were recovered (i.e., 16 in total). The RNA extraction and cDNA synthesis processes were described previously [30]. Total RNA was extracted using the Invisorb^®^ Spin Cell RNA Mini kit (Qiagen, Venlo, The Netherlands). Yolk-sac and allantochorionic membranes and endometrium samples (30 mg) were homogenized separately in 600 µL lysis buffer, and total RNA was eluted with 30 µL Elution buffer. The RNA concentration and integrity were measured using a NanoDrop Spectrophotometer (ND 1000; Isogen Life Sciences, De Meern, The Netherlands) and a BioAnalyzer (Agilant, Palo Alto, CA, USA), respectively. Reverse transcription was performed using Superscript III (Invitrogen) as described previously [31], in a total volume of 20 µL composed of 10 µL of sample containing 1000 ng of RNA, which was treated with DNAse I (30 min at 37 °C followed by 10 min at 65 °C; 1 IU/mg of RNA; RNAse-Free DNase set, Qiagen, Hilden, Germany).

#### 2.5.2. Quantitative RT-PCR

The qRT-PCR protocol was performed as described previously [30], with minor modifications. Primers were produced at Eurogentec (Seraing, Belgium) and specificity was tested by DNA sequencing (ABI PRISM 310 Genetic analyzer; Applied Bio-system, Foster City, CA, USA). Real-time PCR was conducted in 15 µL of reaction mix including 7.5 µL of IQ SYBR^®^ Green Supermix (BioRad, Veenendaal, The Netherlands), 0.075 µL of primer (forward and reverse), and 1 µL of cDNA, on an IQ5 real-time PCR detection system (BioRad; Veenendaal, The Netherlands). Cycle conditions were denaturation for 4 min at 95 °C, followed by 40 cycles of amplification (15 s at 95 °C, 30 s at primer specific annealing temperature and 30 s at 72 °C). For each gene, a melting curve and standard curve were performed to verify product specificity and enable expression quantification. Relative gene expression was expressed as the ratio of target gene expression to the geometric mean of three housekeeping genes (*GAPDH, PGK1* and *SRP14*; Appendix Table A1), selected after stability evaluation using GeNorm [32].

For the conceptus membranes (YS and AC), the expression of 27 candidate genes was determined at day 28 of conceptus development; these included four maternally expressed *(IGF2R, H19, GRB10* and *PHLDA2*) and eight paternally expressed (*INSR, SNRPN, HAT1, DIO3, PEG3, PEG10, IGF2* and *NDN*) genes, three DNA methyltransferase enzymes (*DNMT1, DNMT3A* and *DNMT3B*), four glucose transporters (*SLC2A1, SLC2A3, SLC2A4* and *SLC5A11*), one fructose (*SLC2A5*) and seven amino-acid transporters (*SLC1A1, SLC1A4, SLC1A5, SLC7A2, SLC7A5, SLC38A2* and *SLC43A2*) [18,21]. For the endometrium samples collected from recipient mares immediately after collection of a day-28 conceptus, the expression of four glucose (*SLC2A1, SLC2A3, SLC2A4* and *SLC5A1*) and four amino-acid transporters (*SLC1A4, SLC1A5, SLC7A1* and *SLC38A2*) was determined [18,21].

### 2.6. Glucose Uptake

To quantify glucose uptake capacity, parts of the YS and AC from the sixteen day-28 conceptuses recovered were used for 2-NBDG live-cell staining. Following uterine lavage, the membrane samples were transferred from flushing medium (lactated Ringer’s solution) to 1 mL of PBS at 37 °C in two 4-well plates (YS and AC were cut into two pieces and a part transferred to each of the 4 wells) and washed once with 37 °C PBS. Next, 500 µL of PBS and 5 µL of Hoechst 33342 (5 µg/mL; Sigma-Aldrich Chemical Co., St. Louis, MO, USA) were added to both the control and test wells, and 200 µM of 2-NBDG (ab235976; Abcam, Cambridge, UK; Blodgett et al. [33]) was added to the test well only. The wells were spun to mix the solution thoroughly, and the tissue samples were incubated for 35 min at 37 °C and 5% CO_2_. The solution was then removed, and the tissue samples were washed briefly four times with 37 °C PBS and then immersed once for 5 min. Next, one drop of PBS was introduced into a fluorodish (FD35-100, FluoroDish Cell Culture Dish, World Precision Instruments, Friedberg, Germany) and a tissue sample was transferred to the fluorodish and flattened; a coverslip was placed on top of the tissue sample. Membranes were then used immediately for confocal imaging. Fourteen of the sixteen recovered conceptuses were used for the final analysis (HE-HE group: *n* = 4; C-C group: *n* = 4; C-HE group: *n* = 3; HE-C group: *n* = 3; Table 2), two conceptuses were excluded because the membranes were incomplete or damaged during retrieval.

### 2.7. Lipid Droplets

Following uterine lavage, ten day-7 embryos (5 from control and 5 from HE donor mares) were fixed in 4% paraformaldehyde (PFA; Electron Microscopy Sciences, Hatfield, PA, USA) at 37 °C for 1 h and subsequently stored in 1% PFA at 4 °C for a maximum of one week. Embryos were then placed in 0.1% saponin (1 mg/mL; # 45ZG04; Sigma-Aldrich Chemical Co., St. Louis, MO, USA) in PBS for 30 min at room temperature (RT) to permeabilize the plasma membrane and then incubated in a 0.165 µM solution of Alexa Fluor^®^ 568 phalloidin (Molecular Probes Europe BV, Leiden, The Netherlands) in PBS (1:100 dilution; e.g., 2 µL stock + 198 µL PBS) for 30 min at RT to visualize the actin cytoskeleton. Next, the embryo was washed three times in PBS containing 3% polyvinylpyrrolidone (PBS-PVP). To label fat droplets, the tissue was then incubated in a 1:50 solution of BODIPY 493/503 (D3922, Thermo Fisher, Waltham, MA, USA) in PBS for 60 min in the dark at RT. Next, a washing step in PBS-PVP was performed, and the embryo was then incubated in 0.1 µg/mL 4′,6-diamidino-2-phenylindole (DAPI) in PBS for 30 min in the dark at RT to label the cell nuclei. Subsequently, the embryo was mounted in a 0.12 mm eight-well Secure-Seal Spacer (Molecular Probes) on a glass slide (Superfrost Plus; Menzel, Braunschweig, Germany), covered in Vectashield (10 µL; Vector Laboratories, Burlingame, CA, USA) and sealed with a cover slip using nail polish. For the final analysis, eight of the ten recovered embryos were used, the other two embryos were damaged during processing (Table 3).

### 2.8. Image Acquisition and Analysis

Digital images of stained embryos or conceptus membranes were obtained using a Leica SPE-II confocal microscope equipped with a 20× objective (Leica Microsystems, Wetzlar, Germany). Hoechst 33342 and DAPI were stimulated with a 405-nm laser, and emission was detected between 414 and 466 nm (blue channel). 2-NBDG and BODIPY 493/503 were stimulated with a 488 nm laser, and emission was detected in the 511–577 nm range (green channel). Sequential confocal sections (Z-stacks) were acquired at 2 µm intervals through the whole thickness of the day-7 embryos, or the membranes of day-28 conceptuses. A three-dimensional image of the embryo or membrane was then created and analyzed using Imaris 8.2 software (Bitplane AG, Zurich, Switzerland). The Imaris surface tool was used to render solid surfaces best representing both the lipid droplets, the 2 NBDG uptake and the cell nuclei.

#### 2.8.1. Glucose Uptake

**Figure 2:**
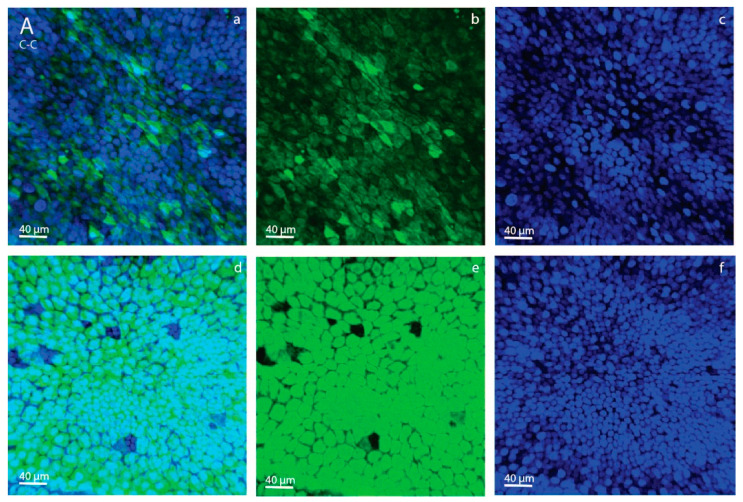

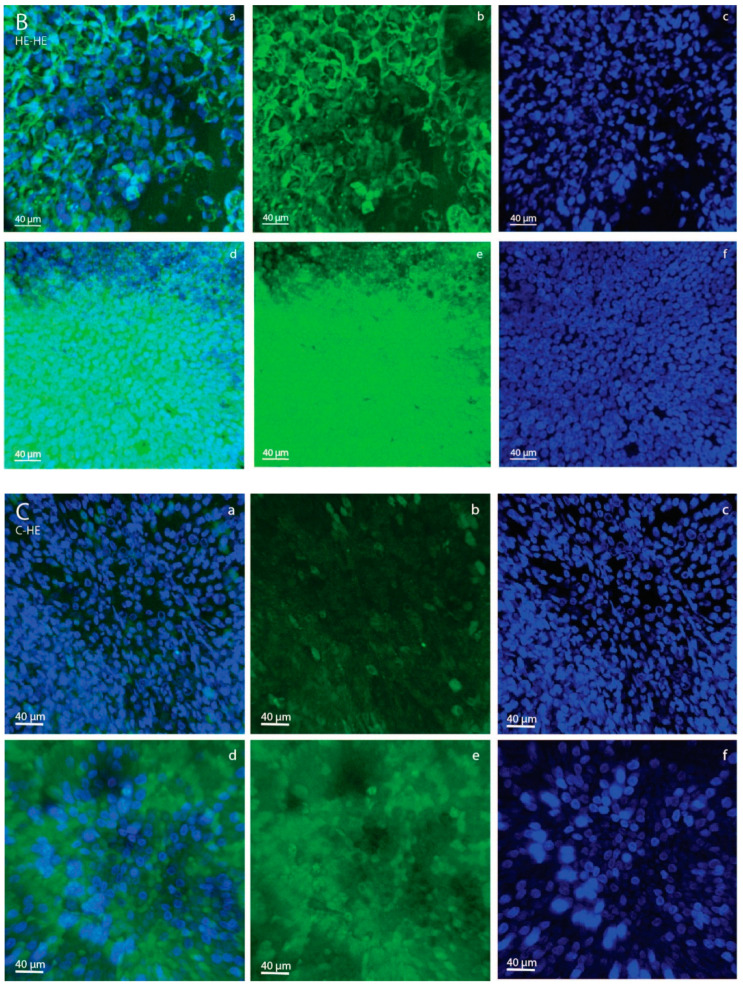

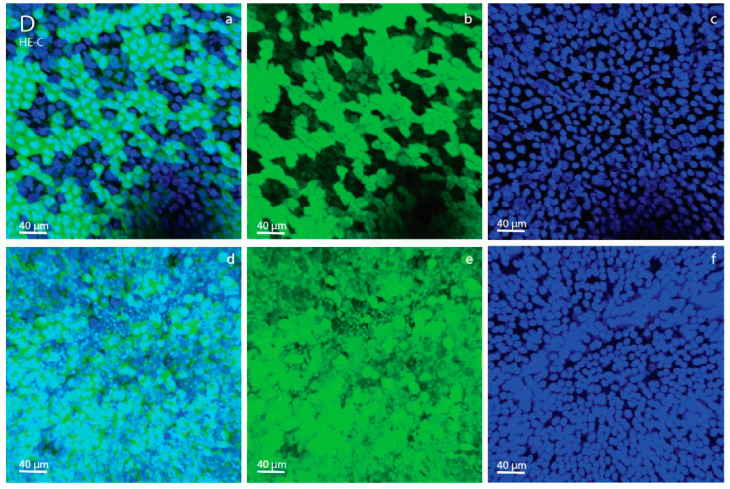
Confocal micrographs of stained allantochorionic (AC; a–c) and yolk-sac (YS; d–f) membranes, obtained using a Leica SPE II DMI-4000 confocal microscope (Leica Microsystems, Wetzlar, Germany), of day-28 embryos that were transferred on day 7 of gestation from a control to a control Shetland pony mare (**A**), high-energy (HE) to HE mare (**B**), control to HE mare (**C**) or HE to control mare (**D**). Cell nuclei are labeled with Hoechst (blue fluorescence). The green fluorescent-stained glucose analog 2-NBDG illustrates glucose uptake by the conceptus membranes. (**a**,**d**): merged micrograph; (**b**,**e**): blue channel (414–466 nm); (**c**,**f**): green channel (511–577 nm).

For the day-28 conceptuses, four different images per tissue sample at 4 different locations on the tissue sample were acquired. Per conceptus, a positive (Hoechst + 2-NBDG) and negative (Hoechst) sample were analyzed for both the YS and AC tissues, producing 16 images per conceptus in total (4 positive and 4 negative YS images, 4 positive and 4 negative AC images). The negative samples were used to estimate background, non-specific fluorescence. The median fluorescence intensity for the negative samples was subtracted from the values for the positive samples to produce a value for specific fluorescence, and median fluorescence intensity for 2-NBDG was calculated per sample. For each ET group, a confocal image of a positive YS and a positive AC sample is shown in Figure 2.

#### 2.8.2. Lipid Droplets

**Figure 3:**
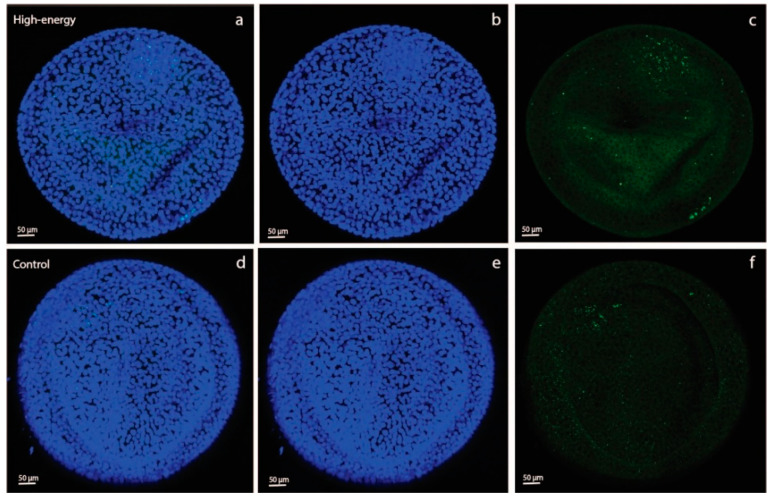
Confocal micrographs of day-7 embryos originating from Shetland pony mares fed a high-energy (200% of net energy requirements; (**a**–**c**)) or control (maintenance; (**d**–**f**)) diet, obtained using a Leica SPE II DMI-4000 confocal microscope (Leica Microsystems, Wetzlar, Germany). Cell nuclei are labeled with 4′,6-diamidino-2-phenylindole (DAPI; blue fluorescence). Lipid droplets are labeled with BODIPY 493/503 (green fluorescence). (**a**,**d**): merged micrograph; (**b**,**e**): blue channel (414–466 nm); (**c**,**f**): green channel (511–577 nm).

For the day-7 embryos, maximum projections through all of the sections were analyzed per embryo. The diameter of each embryo was measured using the length and width axes of the image, and the mean of these two values was calculated. The overall total volume of all fat droplets (total volume of BODIPY fluorescence) was measured and divided by the total number of nuclei for each individual embryo. Confocal images of stained day-7 embryos originating from a control and an HE mare are shown in Figure 3.

### 2.9. Statistical Analysis

Statistical analysis was performed using IBM SPSS Statistics for Windows, version 26 (IBM Corp., Armonk, NY, USA). For all data, normal distribution was confirmed by visual inspection of the Q-Q plot of the residuals. Before analysis, a log_10_ transformation was applied to data that were not normally distributed (PCR data; glucose uptake data in terms of differences in fluorescence intensity between groups) to meet the assumption of normality. Data that remained non-normally distributed after a log transformation were analyzed using a Mann–Whitney nonparametric test and presented as median (+ range) values (glucose uptake data—difference in fluorescence intensity between YS and AC tissue within groups; lipid droplet data). To compare the differences between groups for expression of the imprinted genes and DNMTs in conceptus membranes, a linear mixed model with post hoc Bonferroni testing was used, with donor mare as a random effect and group as a fixed effect. To compare between-group differences in gene expression in the endometrial tissue of recipient mares, in glucose and amino acid transporter expression in the conceptus membranes, and in glucose uptake in both AC and YS, a linear mixed model with recipient mare as a random effect, and group as a fixed effect, was performed. A Benjamini–Hochberg procedure was performed to correct for the testing of multiple genes, using a false discovery rate of 15%. *p* values are presented as raw *p* values (rather than adjusted *p* values); a significant *p* value for a gene expression comparison indicates that it was significant according to Benjamini–Hochberg. Values of *p* < 0.05 were considered significant, unless otherwise stated.

## 3. Results

### 3.1. Body Weight

Body weight (BW) for the control and HE groups is presented for the start and end of each diet year in Table 4 (mean ± SD). Mean BW within the control group was reasonably constant (1% increase during the first year (*n* = 9), 3% during a second year (*n* = 2), and 2% increase during a third year (*n* = 2) on the diet). Mean BW of the HE group increased by 29% during their first year on the diet (*n* = 9), and by 24% during a second year (*n* = 7); BW in HE mares did not decrease markedly during the winter “rest” period and was significantly higher at the start of the second than at the start of the first year or in control ponies.

### 3.2. Gene Expression

#### 3.2.1. Maternally Expressed Genes

**Figure 4:**
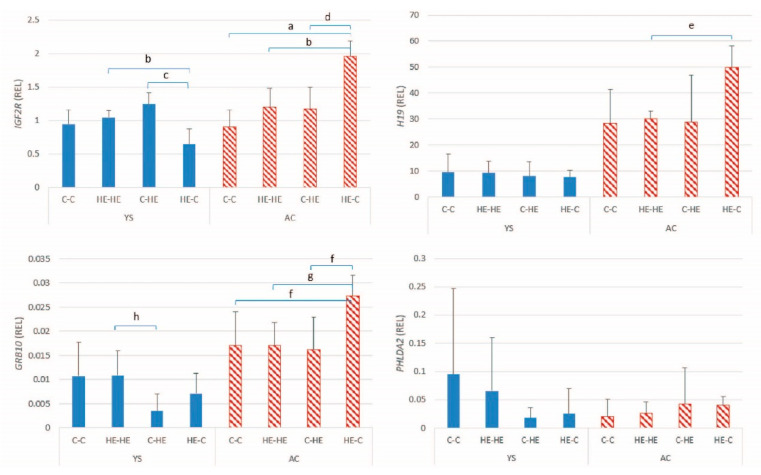
Mean ± SD relative gene expression (REL) for the maternally expressed genes in the yolk-sac (YS) and allantochorionic (AC) membranes at day 28 of conceptus development, after transfer of day-7 embryos from a control to a control Shetland pony mare (C-C; *n*= 4), high-energy (HE) to HE mare (HE-HE; *n* = 4), control to HE mare (C-HE; *n* = 4) or HE to control mare (HE-C; *n* = 4). ^a^ *p* < 0.001; ^b^ *p* = 0.019; ^c^ *p* = 0.004; ^d^ *p* = 0.022; ^e^ *p* = 0.025; ^f^ *p* = 0.033; ^g^ *p* = 0.016; ^h^ *p* = 0.042.

The mean ± SD relative expression for the 4 maternally expressed genes examined in the conceptus membranes is presented in Figure 4. A significant between-embryo transfer group difference was found for the expression of *IGF2R* in AC (*p* = 0.001) and YS (*p* = 0.003), *H19* in AC (*p* = 0.009), and *GRB10* in AC (*p* = 0.006) and YS (*p* = 0.023). Expression was higher in the HE-C group than all other ET combinations. For *PHLDA2*, a significant effect of ET group was found for YS (*p* = 0.037) but post hoc analysis did not detect any significant differences between specific pairs of groups.

#### 3.2.2. Paternally Expressed Genes

**Figure 5:**
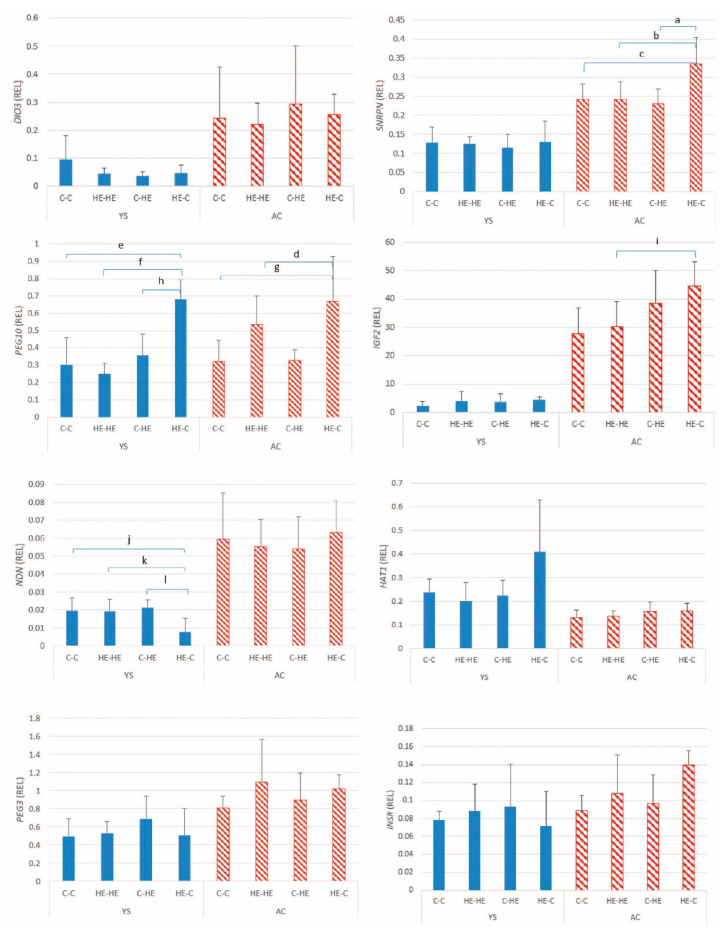
Mean ± SD relative gene expression (REL) for the paternally expressed genes in the yolk-sac (YS) and allantochorionic (AC) membranes at day 28 of conceptus development, after transfer of day-7 embryos from a control to a control Shetland pony mare (C-C; *n* = 4), high-energy (HE) to HE mare (HE-HE; *n* = 4), control to HE mare (C-HE; *n* = 4) or HE to control mare (HE-C; *n* = 4). ^a^ *p*= 0.01; ^b^ *p* = 0.011; ^c^ *p* = 0.026; ^d^ *p* = 0.035; ^e^ *p* = 0.002; ^f^ *p* = 0.001; ^g^ *p* = 0.018; ^h^ *p* = 0.008; ^i^ *p* = 0.047; ^j^ *p* = 0.041; ^k^ *p* = 0.024; ^l^ *p* = 0.041.

For the 8 paternally expressed genes examined in day-28 conceptus membranes, the mean ± SD relative expression is presented in Figure 5. Significant between-group differences were evident for the expression of *PEG10* in YS (*p* < 0.001) and AC (*p* = 0.007), *NDN* in YS (*p* = 0.010), *SNRPN* in AC (*p* = 0.004), and *IGF2* in AC (*p* = 0.015). For most of the genes, the general pattern was the same as that observed for the maternally expressed genes, i.e., higher expression in the HE-C group than in any other combination (Figure 5). For *INSR*, a significant group effect was evident for AC (*p* = 0.034), but post hoc analysis did not identify significant differences between specific pairs of groups. For the other 3 paternally expressed genes, no significant group effects were found (Table 5).

#### 3.2.3. DNA-Methyltransferases

**Figure 6:**
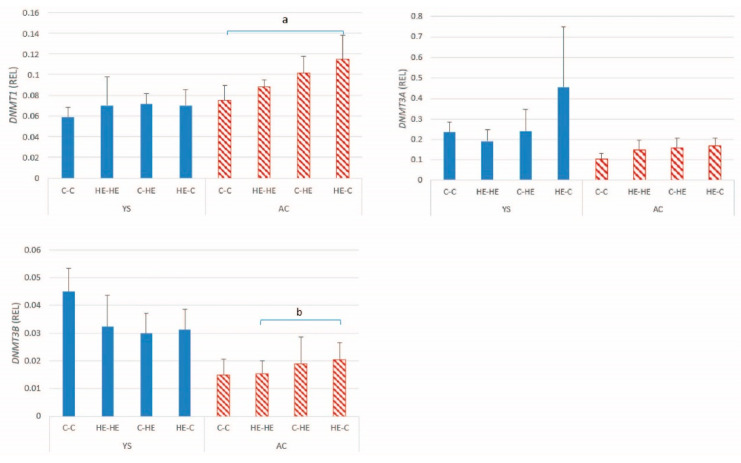
Mean ± SD relative gene expression (REL) for DNA methyltransferases (DNMTs) in the yolk-sac (YS) and allantochorionic (AC) membranes at day 28 of conceptus development, after transfer of day-7 embryos from a control to a control Shetland pony mare (C-C; *n* = 4 high-energy (HE) to HE mare (HE-HE; *n* = 4), control to HE mare (C-HE; *n* = 4) or HE to control mare (HE-C; *n* = 4). ^a^ *p* = 0.01; ^b^ *p* = 0.011.

The mean ± SD relative DNMT expression in conceptus membranes is presented in Figure 6. For *DNMT1* (*p* = 0.007) and *DNMT3B* (*p* = 0.005), a significant between-group difference was found for AC, with the highest expression in the HE-C group, for the maternally and paternally expressed genes. For *DNMT3A*, no significant group effect was apparent (Table 5).

#### 3.2.4. Nutrient Transporters

**Figure 7:**
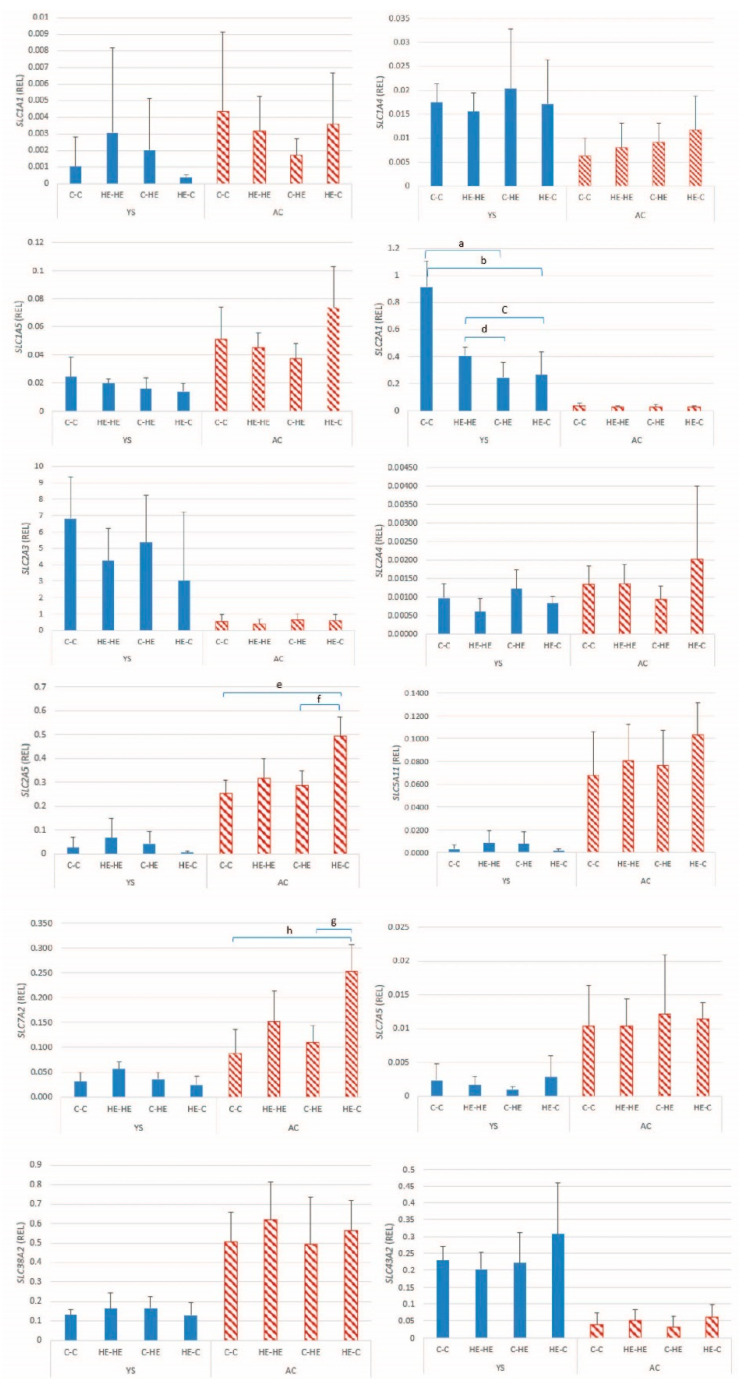
Mean ± SD relative gene expression (REL) for a selection of glucose and amino-acid transporters in the yolk-sac (YS) and allantochorionic (AC) membranes at day 28 of conceptus development, after transfer of day 7 embryos from a control to a control Shetland pony mare (C-C; *n* = 4), high-energy (HE) to HE mare (HE-HE; *n* = 4), control to HE mare (C-HE; *n* = 4) or HE to control mare (HE-C; *n* = 4). ^a^ *p* < 0.001; ^b^ *p* = 0.035; ^c^ *p* = 0.035; ^d^ *p* = 0.03; ^e^ *p* = 0.009; ^f^ *p* = 0.008; ^g^ *p* = 0.035; ^h^ *p* = 0.004.

The mean ± SD relative gene expression for the 5 glucose and 7 amino-acid transporters in day 28 conceptus membranes is presented in Figure 7. For the glucose transporters, a significant difference between ET groups was found for *SLC2A1* expression in YS (*p* < 0.001), and for *SLC2A5* in AC (*p* = 0.001), with highest expression in respectively the C-C and HE-C groups. Among the amino-acid transporters, a significant group effect was found for *SLC7A2* in AC (*p* = 0.004), with expression higher in the HE-C group than in other groups. For *SLC2A3*, a significant overall effect of group was evident for AC (*p* = 0.024), but post hoc analysis did not identify significant differences between specific pairs of groups. For the expression of the other nutrient transporters (i.e., 2 glucose transporters and 6 amino acid transporters), no significant group effects were found (Table 5).

**Figure 8:**
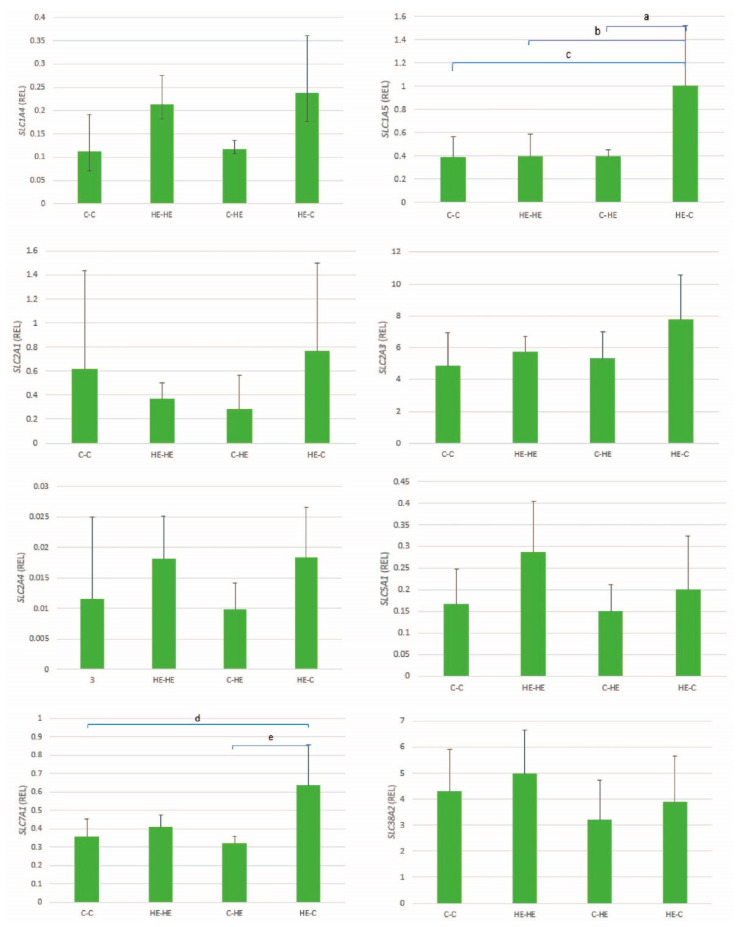
Mean ± SD relative gene expression (REL) for a selection of glucose and amino-acid transporters in the endometrium of recipient Shetland pony mares at day 28 of conceptus development, after transfer of day-7 embryos from a control (C) to a control mare (C-C; *n* = 4), high-energy (HE) to HE mare (HE-HE; *n* = 4), control to HE mare (C-HE; *n* = 4) or HE to control mare (HE-C; *n* = 4). ^a^ *p* = 0.002; ^b^ *p* = 0.003; ^c^ *p* < 0.001; ^d^ *p* = 0.01; ^e^ *p* = 0.003.

For recipient mare endometrium, the mean ± SD relative gene expression for the glucose and amino-acid transporters is presented in Figure 8. No significant differences in expression of glucose transporters were found between groups. For the amino-acid transporters, a significant between-group difference was found for *SLC1A5* (*p* < 0.001) and *SLC7A1* (*p* = 0.002) with, as for a number of the conceptus genes, the highest expression in the HE-C group. For *SLC1A4*, a significant overall group effect was found (*p* = 0.021), but post hoc analysis failed to identify pairwise significant differences between groups. For the other nutrient transporters, no significant group effects were apparent (Table 5).

### 3.3. Glucose Uptake

**Figure 9:**
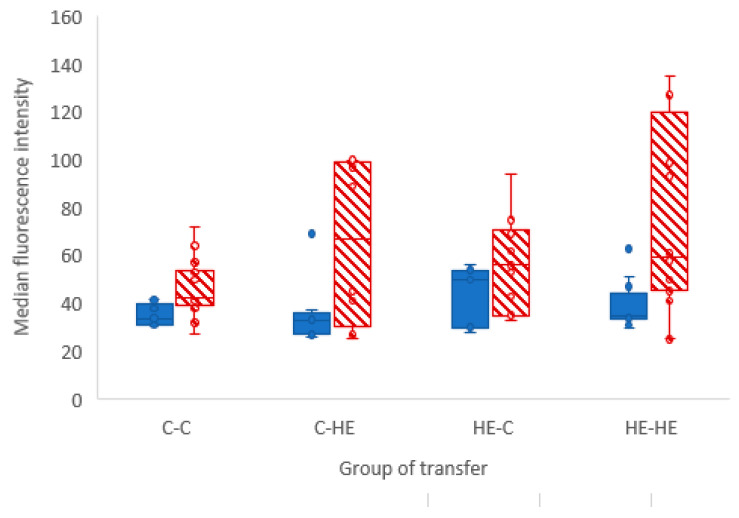
Median (+range) intensity of 2-NBDG fluorescence for the allantochorionic (AC) and yolk-sac (YS) membranes recovered 21 days after transfer of day-7 embryos between Shetland pony mares on different feeding regimes (control to control mare, C-C; control to high-energy mare, C-HE; high-energy to control mare, HE-C; high-energy to high-energy mare, HE-HE.

The median (and range) 2-NBDG intensity values for the pieces of YS and AC membrane are presented in Figure 9. The median fluorescence intensity for all negative control samples was 0. No significant difference between groups was found for either the YS (*p* = 0.7) or AC (*p* = 0.4). With regard to the difference in fluorescence intensity between YS and AC within a group, a significant difference was found for the C-C (*p* = 0.006) and HE-HE (*p* = 0.006) groups, in the form of a higher intensity (i.e., higher glucose uptake) in YS than in AC. This difference was not found in membranes from the C-HE (*p* = 0.21) or HE-C (*p* = 0.069) groups.

### 3.4. Lipid Droplets

**Figure 10:**
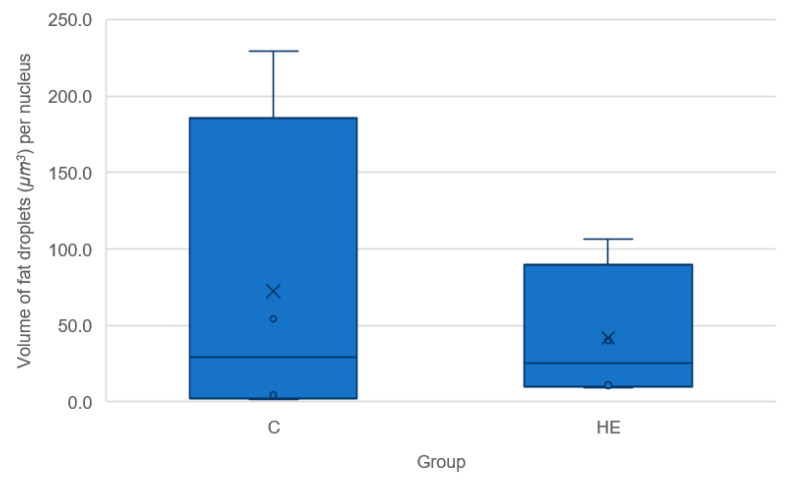
The total volume of fat droplets (µm^3^) divided by the total number of cells for blastocysts recovered form Shetland pony mares fed either a control (maintenance; *n* = 4) or high-energy diet (HE; 200% net energy requirements; *n* = 4).

The total volume of fat droplets (µm^3^) per nucleus (i.e., per cell) for blastocysts recovered from control or HE donor mares is presented in Figure 10. There was no apparent relationship between the diameter of the embryo and the total volume of fat droplets. In addition, there was no difference in the total volume of fat droplets per cell between embryos originating from control or HE donor mares (*p* = 0.77).

## 4. Discussion

This study aimed to examine the effect of maternal overfeeding during the periconception period on nutrient handling and imprinted gene expression in Shetland pony preimplantation conceptuses. To this end, we examined the expression of a range of glucose and amino acid transporters and genes involved in DNA methylation and imprinted genes, in which the latter are thought to be particularly sensitive to epigenetic perturbation during the periconception period. In addition, we examined the actual glucose uptake into the extra-embryonic membranes and lipid accumulation in blastocyst stage embryos and the expression of glucose and amino acid transporters in the endometrium of the recipient mare. Maternal overfeeding was found to affect the expression of three maternally and four paternally expressed genes, two DNMTs, two glucose transporters and one amino acid transporter in the extra-embryonic membranes of day-28 conceptuses and the expression of two different amino acid transporters in the endometrium of recipient mares; in almost all cases, this effect took the form of significantly higher expression when embryos were transferred from an HE donor to a control recipient mare. Conversely, there was no significant effect of maternal overnutrition on embryonic content of lipid droplets on day 7 of gestation or on glucose uptake by the extra-embryonic membranes on day 28 of gestation.

An association was found between maternal overfeeding and the expression of seven imprinted genes in conceptus membranes at day 28 of gestation; these included three maternally expressed (*IGF2R, H19* and *GRB10*) and four paternally expressed genes (*PEG10, NDN, IGF2* and *SNRPN*). The overall pattern was in agreement with previous studies that reported an effect of maternal overfeeding on placental gene expression in mice [15,16]. For example, exposure to a high-sugar, high-fat (HSHF) diet during murine gestation resulted in altered placental expression of a range of imprinted genes and associated changes in metabolic signaling pathways involved in placental growth, metabolism and nutrient transport at day 16 of gestation; this included upregulation of *GRB10*, *IGF2,* and the maternally expressed *H19* gene that is linked to IGF2 expression [16]. Similarly, for murine embryos transferred at the two-cell stage between mice fed either a control or high-fat (HF) diet, both a pre-gestational and gestational HF diet resulted in fetal growth restriction and abnormal placentation [15] and, in the group in which embryos were transferred from HF to control dams, several imprinted genes, including *IGF2* and *IGF2R,* which were differentially expressed in the placenta compared to when embryos were transferred between control dams [15]. For most of the imprinted genes in the present study, the relative expression was higher in the conceptus membranes of embryos transferred from an HE donor to a control recipient mare, irrespective of whether the genes were maternally or paternally expressed; the exception was *NDN*, which showed a drop in expression. That the expression of these genes was higher in the conceptus membranes of embryos originating from an HE donor and transferred to a control maternal environment on day 7 of gestation suggests that maternal overnutrition or obesity has an effect on the oocyte or on the early embryo, as previously proposed in both mice [15] and horses [34,35]. However, there was no increase in expression of imprinted genes in the HE-HE group, although these embryos also originated from an HE donor mare; this is in agreement with the modest differences reported by Sessions-Bresnahan et al. [35] in expression of a range of genes between day 16 conceptuses from obese versus normal mares. Our findings may therefore indicate that the transfer from an excessively nutrient-rich to a “normal” maternal environment results in a mismatch between embryonic expectations and maternal provision and that it is this change that leads to dysregulation of a range of imprinted genes. In this case, early exposure to an HE diet will still need to be responsible for underlying changes that only become apparent when the uterine environment does not meet the altered embryonic requirements. As reported in a previous study, there were no significant between-embryo transfer group differences in crown-rump length or weight of the embryo at day 28 of gestation [36], which presumably indicates that the relative upregulation of the imprinted genes resulted in a better match between the maternal environment and embryonic needs, rather than leading to excessive or restricted embryonic growth. The exact nature of the relationship between maternal overnutrition and the changes in imprinted gene expression can, however, not be determined based purely on our results. Moreover, whether these changes persist during later gestation and into post-natal life has not been established.

Since changes in DNA methylation are likely to play an important role in regulation of gene expression and maintenance or re-establishment of imprinting status, we also examined expression of the DNA methyltransferases. A significant effect of maternal overnutrition was evident for *DNMT1* and *DNMT3B* expression in the AC of day-28 conceptuses. Previous studies in other species have described effects of maternal nutrition on the DNA methylation status of developing conceptuses. For example, in the placenta of obese human mothers, global methylation levels are higher than in the placenta of normal weight mothers at birth [37]. In the present study, a similar pattern for the change in expression of *DNMTs 1* and *3B* was found to be that described for the imprinted genes, namely higher expression in the AC of conceptuses resulting from transfer of an embryo from an HE donor to a control recipient mare. This finding may be reflected by an alteration in the DNA methylation status of embryos transferred from an overfed mare to a neutral maternal environment; however, DNA methylation status was not examined. Conversely, Sessions-Bresnahan et al. [35] compared global DNA methylation status of day-16 equine conceptuses recovered from normal versus obese mares and reported a tendency to higher levels of hydroxy-methylation in embryos from the normal mares, suggesting that the changes we observed in DNMT expression most likely result from the effects of maternal overnutrition (or associated increase in body condition) on the oocyte or early embryo. However, similar to the discussion for imprinted genes, no significant increase in DNMT expression was evident in the HE-HE group, suggesting that any effects at the oocyte level or before day 7 of embryo development only become apparent when the embryo is exposed to an environment that does not meet its expectations or after a longer period of exposure to maternal overnutrition or obesity.

Following its arrival in the uterus at the blastocyst stage of development, the primary energy substrate for the developing embryo is glucose. Moreover, the trophectoderm and extra-embryonic membranes of the pre-implantation equine conceptus express a number of glucose transporters, which help ensure adequate glucose provision to the embryo [18]. In the present study, a significant effect of maternal overfeeding was found on the expression of *SLC2A1* (GLUT-1) in the YS membrane, and on *SLC2A5* (GLUT-5) in AC at day 28 of gestation. As for certain imprinted genes and DNMTs, relative AC *SLC2A5* expression was higher after transfer of embryos from an HE donor to a control recipient mare. In contrast, *SLC2A1* expression was higher in the YS membrane of conceptuses, resulting from transfer of an embryo from a control donor to a control recipient mare, which presumably reflects a down-regulation in the other groups as a response to overfeeding of either the donor or the recipient mare. In the horse, *SLC2A1* has been proposed to play a role in transporting glucose into the yolk sac via facilitated glucose diffusion [18], which explains the relatively high expression detected in the YS membranes in the present study. In the case of maternal overnutrition, downregulation of *SLC2A1* in the YS membrane presumably helps prevent the embryo from being oversupplied with glucose. A previous study showed that the expression of GLUT-1 protein significantly decreased in day-9–10 rat embryos and yolk-sac tissue cultured in medium containing high concentrations of glucose (33.3 and 66.6 mmol/l glucose) for 48 h [38]. The expression of GLUT-1 mRNA was, however, not downregulated in that study. GLUT-1 is a fructose transporter [39], and upregulation of expression in the AC of the HE-C group may reflect an imbalance between the fructose needs of an embryo that initially developed in an HE donor mare and presumed lower supply of fructose in the uterine environment of a control recipient mare.

For the amino acid transporters, only *SLC7A2* in the AC of embryos transferred from an HE donor to a control recipient mare showed an altered pattern of expression, namely upregulation. *SLC7A2* has previously been shown to be upregulated in equine conceptus membranes as early pregnancy proceeds and has been suggested to help delivery of amino acids from the uterine lumen to the trophectoderm [21]. The relatively high expression of *SLC7A2* may reflect an imbalance between amino acid demands of an embryo that initially developed in an HE environment and supply in the control uterine environment.

The general findings for nutrient transporter expression are in agreement with other studies, namely an increase in expression of certain amino acid and glucose transporters in the trophoblast membranes and early placentae of mice fed an HF [22] or HSHF [23] diet. However, Sasson et al. [15] reported that the expression of the upregulated transporters normalized during later pregnancy and concluded that early changes in nutrient transporter expression are neither permanent nor responsible for differences in the metabolic phenotype. Moreover, they noted that there was no proof that upregulation of transporter expression necessarily resulted in a change in actual nutrient transport [15]. Similarly, Sferruzzi-Perri et al. [16] showed that while a maternal HSHF diet resulted in growth restriction of mouse fetuses on day 16 of gestation, this growth restriction was no longer evident by day 19; moreover, the expression of the nutrient transporters was unaffected, again, suggesting that temporal differences in the embryonic and fetal expression of genes encoding for nutrient transporters induced by maternal periconception overnutrition do not become permanent alterations. In the present study, we investigated glucose uptake by day-28 conceptus membranes and found no significant effect of maternal nutrition status on the uptake of the fluorescent glucose analog 2-NBDG, which is thought to be transported by both sodium-glucose linked transporters (SGLTs) and glucose transporters (GLUTs) [33]. However, fructose uptake was not measured, and it cannot be determined whether maternal overfeeding affected fructose uptake via GLUT-5. The glucose analogue used in our study could have been taken up into the day-28 membranes via GLUT-5, but based on findings in other species, it cannot be confirmed or ruled out, and we suggest that GLUT-5 did not play a significant role in glucose analogue absorption [39]. If the increase in gene expression for the fructose and amino acid transporters did not lead to increased uptake of glucose, fructose and amino acids, it may also help explain why maternal nutritional status did not affect embryo size or weight at day 28 of gestation [36]. If few amino acid transporters are affected (only one of the seven we examined), the change is unlikely to have significant effects on the whole system since there is a large degree of overlap and redundancy among the amino acid transporters, and up- or down-regulation of the majority within a type (neutral, anionic, cationic) will be required to significantly impact overall amino acid transport [40]. In a similar vein, the lipid droplet content did not differ between blastocysts originating from control or HE donor mares, indicating that maternal overfeeding had no gross effect on lipid uptake and storage prior to day 7 of gestation.

As an aside, we saw a difference in 2-NBDG fluorescence intensity between YS and AC in both the C-C and HE-HE groups, with a higher intensity in YS than AC (Figure 2). This suggests that, in ponies, the day-28 YS membrane had a greater capacity to take up glucose than the AC. This presumably suggests that the YS is still the primary route of nutrient uptake, whereas the AC is not yet fully developed, e.g., has yet to sufficiently attach to and interact with the endometrium to take up its role as the major route for nutrient supply.

Another important potential contributor to the abundance of nutrients in the uterine lumen is the expression of nutrient transporters in the endometrium, and the expression of amino acid transporters in the mare’s endometrium has been shown to change during early pregnancy, presumably as a result of the presence of a conceptus and factors secreted by that conceptus [21]. We found an upregulation of endometrial expression of the amino acid transporters *SLC1A5* and *SLC7A1* in control mares that received an embryo from an HE donor mare. Previously, the expression of *SLC1A5* was reported to be upregulated in equine endometrium between days 14 and 21 of gestation [21], and the transporter is thought to play a crucial role in delivering amino acids to the uterine lumen [21]. *SLC7A1* has previously been reported to be downregulated during early pregnancy [21]. Upregulation of both these amino acid transporters in the endometrium of control mares that received an embryo from an HE donor may reflect an embryo-directed effect to help meet demands for amino acids.

Due to the small number of donor and recipient mares included in this study, several were used twice within a single embryo transfer group and several mares were used during more than one study year, whereas others were used for one year only. Although statistical methods were selected to consider individual mare effects, it is possible that reuse of ponies within groups and over multiple years could have biased the results. Finally, embryos were not divided by sex (male versus female), whereas it is possible that embryos of different genders respond differently to maternal overfeeding; in this respect, Sessions-Bresnahan et al. [35] reported a tendency for a higher level of global methylation in female but not male embryos recovered from normal weight, compared to obese mares.

In conclusion, transferring embryos from overfed Shetland pony mares to recipient mares on a maintenance diet resulted in increased expression of several imprinted genes (both maternally and paternally expressed genes), *DNMTs 1* and *3B*, and a number of glucose and amino acid transporters in the conceptus membranes on day 28 of gestation. Transfer of an embryo from an overfed donor to a control recipient mare also led to upregulated expression of two different amino acid transporters in the endometrium of the recipient mare. Although transferring horse embryos from overfed to control mares seems to disturb gene expression, it did not affect glucose uptake by the conceptus membranes on day 28 of gestation, and it remains to be seen whether the alterations in gene expression are maintained throughout gestation and into postnatal life.

## Figures and Tables

**Table 1:** Composition of the subgroups (C-C, C-HE, HE-C and HE-HE), including the names of the individual ponies, their age, and the year and season in which embryo transfer and conceptus recovery were performed.

Donor Mare	Age	Recipient Mare	Age	Year of Embryo Transfer	Season of Embryo Transfer
C (Bontje)	7	C (Geke)	2	2	Summer
C (Gwyneth)	2	C (Florance)	3	2	Autumn
C (Elivia)	5	C (Bontje)	8	3	Autumn
C (Donja)	5	C (Bontje)	7	2	Summer
HE (Bonte Vos)	8	HE (Avasha)	8	2	Spring
HE (Fabiene)	3	HE (Avasha)	8	2	Autumn
HE (Frederika)	4	HE (Blacky)	5	3	Summer
HE (Andrea)	4	HE (Frederika)	3	2	Autumn
C (Elivia)	4	HE (Gondar)	6	2	Spring
C (Donja)	5	HE (Fabiene)	2	2	Summer
C (Elivia)	5	HE (Dieuwertje)	6	3	Summer
C (Fairview)	2	C (Frederika)	3	2	Spring
HE (Gondar)	6	C (Marion)	8	2	Spring
HE (Andrea)	4	C (Elivia)	4	2	Summer
HE (Avasha)	8	C (Donja)	5	2	Summer
HE (Bonte Vos)	9	C (Bontje)	8	3	Autumn

**Table 2:** Composition of the subgroups (C-C, C-HE, HE-C and HE-HE), including the names of the individual ponies, their age, and the year and season in which embryo transfer and conceptus recovery was performed.

Donor Mare	Age	Recipient Mare	Age	Year of Embryo Transfer	Season of Embryo Transfer
C (Bontje)	7	C (Geke)	2	2	Summer
C (Elivia)	5	C (Bontje)	8	3	Autumn
C (Donja)	5	C (Bontje)	7	2	Summer
C (Gwyneth)	2	C (Florance)	3	2	Summer
HE (Fabiene)	8	HE (Avasha)	8	2	Autumn
HE (Frederika)	4	HE (Blacky)	5	3	Summer
HE (Andrea)	4	HE (Frederika)	3	2	Summer
HE (Andrea)	4	HE (Gondar)	6	2	Autumn
C (Elivia)	5	HE (Dieuwertje)	6	3	Summer
C (Donja)	5	HE (Fabiene)	2	2	Summer
C (Fairview)	2	HE (Frederika)	3	2	Spring
HE (Andrea)	4	C (Elivia)	4	2	Summer
HE (Gondar)	6	C (Marion)	8	2	Spring

**Table 3:** Donor mares including their age and the year and season in which embryo collection was performed.

Donor Mare	Age	Year of Embryo Collection	Season of Embryo Collection
C (Bontje)	5	1	Spring
C (Rosa)	4	1	Spring
C (Elivia)	3	1	Summer
C (Rosa)	4	1	Summer
HE (Gondar)	5	1	Spring
HE (Andrea)	3	1	Spring
HE (Cora)	4	1	Summer
HE (Cora)	5	1	Summer

**Table 4:** Mean ± SD weight (kg) of Shetland pony mares (total *n* = 18) at the start and end of the first, second and third years that were fed a control (maintenance) or a high-energy (200% of net energy requirements) diet.

	Number of Ponies		Body Weight (kg)			
			Control		High-Energy	
Number of Years Consuming the Diet	Control	High-Energy	Start	End	Start	End
One	9	9	176 ± 17	178 ± 16	170 ± 24	219 ± 30
Two	2	7	174 ± 7	169 ± 1	207 ± 27	258 ± 35
Three	2	0	176 ± 4	179 ± 5	-	-

**Table 5:** *p* values for the difference (main group effect) in expression for selected genes in allantochorion and yolk-sac membranes and endometrial tissue recovered from Shetland pony mares on day 28 of gestation following transfer of embryos between overfed and control mares on day 7 after ovulation. *p* values < 0.05 are indicated by a *.

Gene	Allantochorion	Yolk Sac	Endometrium
Maternally expressed genes			
*IGF2*	0.015	0.198	-
*H19*	0.009 *	0.875	-
*GRB10*	0.006 *	0.023 *	-
*PHLDA2*	0.320	0.037 *	-
Paternally expressed genes			
*SNRPN*	0.004 *	0.648	-
*IGF2R*	0.001 *	0.003 *	-
*INSR*	0.034 *	0.769	-
*PEG3*	0.543	0.459	-
*PEG10*	0.007 *	< 0.001 *	-
*NDN*	0.884	0.010 *	-
*HAT1*	0.403	0.067	-
*DIO3*	0.027 *	0.447	-
DNA methyltransferases			
*DNMT1*	0.007 *	0.651	-
*DNMT3A*	0.12	0.131	-
*DNMT3B*	0.005 *	0.073	-
Glucose transporters			
*SLC2A1*	0.780	<0.001 *	0.143
*SLC2A3*	0.398	0.024 *	0.241
*SLC2A4*	0.542	0.090	0.127
*SLC5A11*	0.388	0.701	
*SLC5A1*	-	-	0.200
Fructose transporter			
*SLC2A5*	0.001 *	0.596	-
Amino acid transporters			
*SLC1A1*	0.856	0.795	-
*SLC1A4*	0.372	0.980	0.021 *
*SLC1A5*	0.108	0.337	< 0.001
*SLC7A2*	0.004 *	0.287	-
*SLC7A5*	0.950	0.454	-
*SLC38A2*	0.331	0.555	0.288
*SLC43A2*	0.422	0.681	-
*SLC7A1*	-	-	0.002 *

## Data Availability

The data presented in this study are available in Effect of overfeeding Shetland pony mares on embryonic glucose and lipid accumulation, and expression of imprinted genes, D’ Fonseca et al. 2021.

## Appendix A

**Table A1:** Nucleotide sequences of primers for candidate reference (“housekeeping”) genes used for normalization of quantitative reverse transcription polymerase chain reaction data.

Gene	Sequence	Ta (°C)	Amplicon Size (bp)	GenBank Accession No.
*GAPDH*	Forward: 50 -AGGCCATCACCATCTTCCAG-30	53	112	NM_001163856.1
	Reverse: 50 -CCAGCCTTCTCCAAGGTAGT-30			
				
*PGK1*	Forward: 50 -CTGTGGGTGTATTTGAATGG-30	54	151	XM_005614287.1
	Reverse: 50 -GACTTTATCCTCCGTGTTCC-30			
*SRP14*	Forward: 50 -CTGAAGAAGTATGACGGTCG-30	55	101	XM_001503583.3
	Reverse: 50 -CCATCAGTAGCTCTCAACAG-30			
				

Ta, annealing temperature; *GAPDH*, glyceraldehyde-3-phosphate dehydrogenase; *PGK1*, phosphoglycerate kinase 1; *SRP14*, signal recognition particle 14 kDa.
